# Frequency of Transducer-like Enhancer of Split 1 Immunohistochemical Expression in Synovial Sarcoma: An Institution-based Cross-sectional Study

**DOI:** 10.7759/cureus.6357

**Published:** 2019-12-11

**Authors:** Madiha B Qureshi, Nasir Uddin, Muhammad Usman Tariq, Ahmed Raheem, Shahid Pervez

**Affiliations:** 1 Pathology and Laboratory Medicine, Aga Khan University Hospital, Karachi, PAK; 2 Pathology and Laboratory Medicine, Aga Khan University Hospital , Karachi, PAK

**Keywords:** synovial sarcoma, monophasic synovial sarcoma, poorly differentiated synovial sarcoma, tle1

## Abstract

Background

Soft-tissue sarcomas comprise a diverse group of sarcomas with characteristic histologic features. However, histology alone is not adequate for a definitive diagnosis for many tumors. In such cases, immunohistochemistry (IHC) plays a key role in determining the line of differentiation and exact characterization. Transducer-like enhancer of split 1 (TLE1) has been recently described as a novel marker for synovial sarcoma (SS). Its high sensitivity and specificity make it a potential marker that distinguishes SS from histologic mimics such as malignant peripheral nerve sheath tumor (MPNST), Ewing's sarcoma (ES), and fibrosarcomatous dermatofibrosarcoma protuberans (FS-DFSP). The objective of our study was to assess the frequency of TLE1 immunohistochemical expression on SS cases of various subtypes.

Methods

This cross-sectional study was conducted at the Department of Histopathology, Aga Khan University, Karachi, Pakistan from February 3, 2018 to February 10, 2019. Tissue samples of 89 SS cases were selected for this study. Tumor sections were stained with hematoxylin and eosin (H&E), cytokeratin AEI/AE3 (CKAE1/AE3), epithelial membrane antigen (EMA), and TLE1 immunohistochemical stain. TLE1 expression was assessed based on the Remmele scoring system.

Results

Tissue samples of 89 SS cases were processed for the study. Mean (±) standard deviation (SD) of age was 25 (±7.36) years. Male:female ratio was 1.1:1. Of the 89 SS cases, 42 (47.2%) were monophasic, six (6.7%) were biphasic, and 41 (46.1%) were poorly differentiated. All the 89 cases showed positivity for TLE1 immunostain: 86 (96.6%) cases showed strong positivity, one (1.1%) case showed moderate expression, and two (2.2%) showed weak positivity.

Conclusion

This study shows that TLE1 is a highly sensitive immunostain for SS irrespective of the histologic type. However, it may show weak-to-moderate staining in poorly differentiated types. No statistically significant association was seen with respect to age group, gender, or type of SS.

## Introduction

Synovial sarcoma (SS) is the fourth most common sarcoma, comprising 10% of all soft-tissue sarcomas [1-3]. It has a relatively higher occurrence in the 15-49 age group in Karachi [4]. Most of these tumors (80%) arise in the deep intramuscular soft tissues of extremities, especially around the knee, ankle, feet, and hand. The other commonly affected sites include the inguinal region, abdominal wall, and head and neck. Virtually every site has been reported including nerve, heart, lung, prostate, kidney, etc. [5-6].

Three histologic types of SS are known: monophasic (50-60%), which consists of monomorphic spindle-shaped cells arranged in sheets or fascicles and rare mitosis with differentials of malignant peripheral nerve sheath tumor (MPNST), fibrosarcomatous dermatofibrosarcoma protuberans (FS-DFSP), etc.; biphasic (20-30%), which consists of both spindle and epithelial components; and poorly differentiated SS (10-15%), which consists of diffuse sheets of small round blue cells with nuclear atypia, conspicuous nucleoli, and high mitotic rate with close differential of Ewing's sarcoma (ES) [7-10].

The tumor is characterized by a t(X;18) balanced translocation, which results in a fusion of the SSX gene present on chromosome X to the SYT gene on chromosome 18 [1-11]. The SYT-SSX fusion oncoprotein causes transcriptional dysregulation by repression of tumor-suppressor genes [12,13]. Hence, the gold standard for SS diagnosis is fluorescence in situ hybridization (FISH), reverse transcriptase-polymerase chain reaction (RT-PCR), or cytogenetics. However, unfortunately, in a developing country like Pakistan, the lack of well-equipped laboratories, skilled personnel, and financial constraints limit their use [1,2,14].

Traditionally, a panel of immunohistochemical stains (none of which is specific) including epithelial membrane antigen (EMA), cytokeratins (CK AE1/AE3, CK7, CK19), cluster of differentiation (CD99, CD34), B cell lymphoma 2 (BCL-2), and vimentin are used for SS diagnosis [1,13,15]. Recent studies have shown Transducer-like enhancer of split 1 (TLE1) to be more sensitive and specific than all the other biomarkers in diagnosing and differentiating SS from histologic mimics [1,3]. TLE1 is a member of the Groucho/TLE gene family and encodes a transcriptional protein necessary for hematopoiesis and cellular differentiation [16,17]. TLE1 protein is also involved in the Wnt/β-catenin signaling pathway associated with SS, where it replaces TLE1-TCT/LEF complexes that repress transcription [18-20]. Genetic studies have identified TLE1 as a robust biomarker for differentiating SS from its histologic identicals [21-23].

Several studies worldwide have demonstrated variable sensitivities ranging from 75-95.2% for TLE1 as an immunomarker for SS, with two of them concluding it to be more sensitive for the poorly differentiated subtype [2,3,13,18].

No prior research work has been done in our part of the world to assess the diagnostic utility of TLE1 in SS. Also, relative TLE1 expression in different subtypes needs to be thoroughly studied for differentiating these subtypes from their histologic mimics on an immunohistochemistry (IHC) basis. This study will evaluate the expression of TLE1 in a cohort of SS cases including all subtypes in routine diagnostic practice.

## Materials and methods

The study was performed at the Section of Histopathology, Department of Pathology and Laboratory Medicine, Aga Khan University Hospital, Karachi. All diagnosed SS cases received as incisional tumor biopsies or surgical resection specimens were included in the study. These specimens included both genders of age ranging between 15-35 years, and with tumors from any site of the body. Consent was taken from the parents of patients in the pediatric age group. The diagnosis was given based on classic morphology and positive IHC of immunostains cytokeratin AE1/AE3 and EMA. Poorly fixed or autolyzed specimen (specimen not received in 10% buffered formalin), tru-cut biopsy (TCB) specimens, cases with the diagnosis that says “suggestive of”, and cases diagnosed with other differentials were excluded. Testing was performed on biopsied tissues. Formalin-fixed, paraffin-embedded tissue sections with a thickness of four micrometers were deparaffinized using xylene, rehydrated via dehydrated alcohols, and treated with 10 mM citrate buffer at pH 6.0 at 450 watts in the microwave for 20 minutes to retrieve the antigen. Next, the sections were placed under tap water and the application of hydrogen peroxide blocker was done for up to five minutes in order to block endogenous enzyme peroxidase activity. Distilled water was used for washing slides and the primary antibody, Cell Marque TLE1 monoclonal antibody (Sigma-Aldrich Co. LLC, St. Louis, Missouri), was administered at a dilution of 1:100 for 30 minutes at room temperature. After that, the slides were washed with wash buffer, and polymer labeled with enzyme HRP was applied for 30 minutes at room temperature. Immunostaining was performed using Dako Envision FLEX method with three-diaminobenzidine (DAB) as a chromogen for 10 minutes. Hematoxylin solution was used for counterstaining. Positive known control, as well as the negative control (wash buffer to take the place of the first antibody), was included and run with each batch of IHC staining. Finally, slides were dried in an oven and mounted with mounting media (DPX).

Dark brown nuclear staining was the only result interpreted as positive. The cases were initially analyzed and scored by the principal investigator, and findings were reviewed and confirmed by a consultant histopathologist. Variables including age, sex, patient identification, specimen type, diagnosis (SS type), and immunohistochemical marker positivity were obtained on a predesigned proforma. For IHC staining analysis, the stained slides were examined under a light microscope to look for proportion and intensity of staining according to the Remmele score (0-12) as follows [3]: Remmele score = intensity of immunoreactivity × percentage of the stained tumor cells.

Intensity of immunoreactivity for the Remmele Score was assessed as follows: zero for no staining, one for weak staining (faint light-brown staining), two for moderate/intermediate staining (dark-brown nuclear staining of intensity less than that of positive control), and three for strong staining (dark-brown nuclear staining with similar intensity of positive control). Percentage of positively stained cells was assessed as follows: zero for no staining, one for <10% of the cells staining, two for 11-50% of the cells staining, three for 51-80% of the cells staining, and four for 81-100% of the cells staining. The cumulative score was interpreted as follows: five to twelve = high score, three to four = moderate score, one to two = weak score, and zero = negative score. High and moderate scores were considered as positive TLE1 staining, whereas weak score was considered negative.

Data were entered and analysis was done via Statistical Package for the Social Sciences (SPSS), version 21.0 (IBM, Armonk, NY). Descriptive statistics were determined. Mean (±) standard deviation (SD) was assessed for quantitative variables such as the age of the patient and the Remmele score. Frequency and percentages were also calculated for qualitative variables such as gender distribution, SS type (monophasic, biphasic, and poorly differentiated), and TLE1 IHC staining (yes/no). Effect modifier was kept under control through stratification with regards to age, gender, and type of SS to measure the outcome on TLE1. A chi-square test was utilized, and a probability value (p) of ≤0.05 was considered significant.

## Results

Tissue samples of 89 SS cases were selected for the study. Mean (±) SD of age was 25 (±7.36) years. Male:female ratio was 1.1:1. Of the 89 patients, 47 (53%) were female and 42 (47%) were male.

Of all the 89 SS cases, 42 (47.2%) were monophasic, six (6.7%) were biphasic, and 41 (46.1%) were of poorly differentiated type. The most common site of tumor occurrence was thigh (13 cases), followed by leg (12), and arm (nine). Other sites included the knee, foot, forearm, neck, and buttocks, etc. (Table 1).

**Table 1:** Site distribution of tumors

Site	Frequency (f)	Percentage (%)
Posterior aural	1	1.10
Lymph node	1	1.10
Unknown/not specified	2	2.20
Leg	12	13.50
Neck	5	5.60
Face	3	3.40
Arm	9	10.10
Temporal/extradural	2	2.20
Chest	4	4.50
Knee	8	9.00
Hand	2	2.20
Forearm	6	6.70
Kidney	1	1.10
Popliteal	2	2.20
Groin	1	1.10
Foot	8	9.00
Buttock	4	4.50
Retroperitoneal	1	1.10
Vocal cord	1	1.10
Pelvic	1	1.10
Pleura	1	1.10
Thigh	13	14.60
Lung	1	1.10
Total	89	100

**Figure 1:**
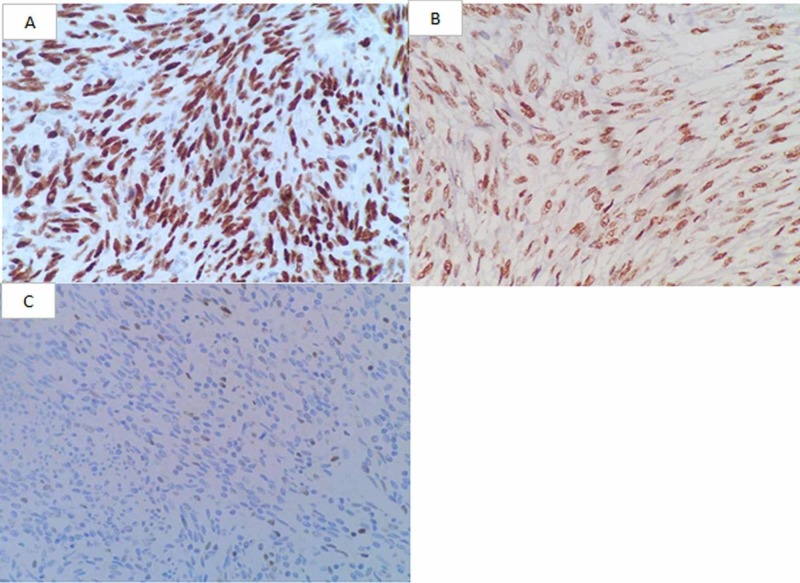
TLE1 immunostaining (A) Strong nuclear TLE1 immunostaining in monophasic type synovial sarcoma, H&E, 20x (B) Moderate/intermediate nuclear TLE1 immunostaining in monophasic type synovial sarcoma, H&E, 20x (C) Weak nuclear TLE1 immunostaining in monophasic type synovial sarcoma, H&E, 20x

The percentage of immunohistochemically positive cells was noted for each case (Table 2). Intensity of immunoreactivity for TLE1 immunostain was recorded for each case (Table 2 and Figure 1). The Remmele score was calculated as described and was found to be equal to and in between two and twelve. Hence, all the SS cases were interpreted as positive for TLE1 immunostain.

**Table 2:** TLE1 immunohistochemical expression TLE1: transducer-like-enhancer of split 1

TLE1 positive cells	Frequency (f)	Percentage (%)
<10% of cells staining	1	1.10
11-50% of cells staining	6	6.70
51-80% of cells staining	14	15.70
81-100% of cells staining	68	76.40
Total	89	100
Intensity of TLE1 staining		
Weak positive	2	2.20
Intermediate positive	1	1.10
Strong positive	86	96.60
Total	89	100

Since all the cases showed TLE1 immunopositivity, effect modifier was kept under control via stratification with regards to age, gender, and type of SS to measure the outcome on TLE1 as strong positive, moderate/intermediate positive, and weak positive. Three age groups were thus made: 15-20 years, 21-30 years, and 31-35 years.

Out of the 89 cases, 32 (36%) were aged 31-35 years, 31 (34.8%) were aged 21-30 years, and 26 (29.2%) were aged 15-20 years. TLE1 interpretation with respect to age groups, gender, and type of SS is shown (Table 3). 

**Table 3:** TLE1 interpretation based on age, gender, and synovial sarcoma type TLE1: transducer-like-enhancer of split 1

Study variables	TLE1 interpretation				P-value
	Negative	Weak positive	Intermediate positive	Strong positive	
Age groups					
15-20 years	0 (0%)	0 (0%)	0 (0%)	26 (29.2%)	0.615
21-30 years	0 (0%)	1 (1.1%)	0 (0%)	30 (33.7%)	
31-35 years	0 (0%)	1 (1.1%)	1 (1.1%)	30 (33.7%)	
Gender					
Male	0 (0%)	2 (2.2%)	0 (0%)	40 (44.9%)	0.207
Female	0 (0%)	0 (0%)	1 (1.1%)	46 (51.7%)	
Synovial sarcoma type					
Monophasic	0 (0%)	0 (0%)	0 (0%)	42 (47.2%)	0.458
Biphasic	0 (0%)	0 (0%)	0 (0%)	6 (6.7%)	
Poorly differentiated	0 (0%)	2 (2.2%)	1 (1.1%)	38 (42.7%)	

## Discussion

It is quite challenging to distinguish SS from histologic mimics on morphology alone [16]. The immunostains currently available including CK AE1/AE, EMA, the cluster of differentiation (CD99, CD34), B-cell lymphoma 2 (BCL-2), and vimentin are less sensitive and specific [12,18]. Genetic analysis has proved TLE1, a gene that exhibits increased expression in SS, to be a remarkable biomarker [17]. There have been several works of research in different parts of the world assessing TLE1 expression in SS with differential expression in occasional studies only. No data have been published in Pakistan on the relevant subject. In this study, the age range is comparable to studies done internationally. The sample size of the study included both incisional tumor biopsies and surgical resection specimens.

In comparison to a study by Knosel et. al., which showed 96% positivity for TLE1, this study has shown 100% TLE1 expression [3]. Strong and moderate TLE1 staining was observed in 97.7% of the cases, whereas weak expression was seen in 2.2%, in comparison to the 75% strong and moderate positive and 21% weak positive cases in the study by Knosel et. al. Another study done by Jagdis et al. showed strong TLE1 positivity in all cases of poorly differentiated SS, which is different from the findings of this study in which only 3.3% of poorly differentiated SS cases showed weak and moderate staining [2]. In the one case of biphasic SS in the study by Jagdis et al., TLE1 was strong positive in both spindle and epithelial cells and this finding is similar to our study.

In another study by Foo et al., 82% of SS cases displayed positivity for TLE1, including 78% biphasic, 79% monophasic, and 91% poorly differentiated types [16]. Similarly, in a study by Chuang et al., 78-86% of SS cases showed nuclear TLE1 expression [1]. This again is in contrast with the current study, which showed 100% TLE1 expression.

Limitations that are conceivable in this study include a lack of financial resources for performing t(X;18) translocation for each case. A comparison of TLE1 expression in other sarcomas would provide much greater insight into establishing the specificity of this robust marker. As far as the findings of this study are concerned, TLE1 is an extremely sensitive immunomarker for SS, irrespective of the type. The greater the staining intensity, the higher the sensitivity.

## Conclusions

This study shows that TLE1 has a high sensitivity for SS as an immunomarker irrespective of the histologic type. However, it may show weak-to- moderate staining in poorly differentiated types of SS. No statistically significant association was seen with respect to age group, gender, or SS type. 
